# Associations Between Nightly Sleep and Daily Activity Diversity Among Older Adults

**DOI:** 10.1093/geroni/igaf122.1602

**Published:** 2025-12-31

**Authors:** Soomi Lee, Sandra Duezel, Denis Gerstorf, Ilja Demuth, Ulman Lindenberger, Rachel Koffer, Johanna Drewelies

**Affiliations:** The Pennsylvania State University, University Park, Pennsylvania, United States; Charité - Universitätsmedizin Berlin, Berlin, Berlin, Germany; Humboldt University Berlin, Berlin, Berlin, Germany; Charité - Universitätsmedizin Berlin, Berlin, Berlin, Germany; Max Planck Institute for Human Development, Berlin, Berlin, Germany; Arizona State University, Phoenix, Arizona, United States; Humboldt University Berlin, Berlin, Berlin, Germany

## Abstract

Greater activity diversity—broad and even participation across daily activities—is found to promote psychological well-being, cognitive functioning, and brain health. Sleep is a key lifestyle factor important for health, but it often competes for time with daily activities. Little is known about its relationship with daily activity diversity in older adults. This study examined the bidirectional associations of nightly sleep quantity and quality with daily activity diversity, as well as age-related differences in these associations. Data came from 127 retired older adults (Mage=76.88, Range=67–88) who participated in a 7-day experience sampling study (6 times/day) embedded into the Berlin Aging Study II. Participants reported momentary engagement in 16 activities across 7 domains (i.e., social, physical, mental, productive, household chores, self-care, and leisure), which were used to calculate a daily activity diversity score using Shannon’s entropy. They also reported nightly sleep duration and quality. Multilevel models, adjusted for age, gender, education, morbidity, perceptual speed, negative affect, and daily pain, tested bidirectional associations between nightly sleep and daily activity diversity. Results showed no significant main association between previous night’s sleep duration/quality and the next-day activity diversity, or vice versa. However, age moderated the association between prior night’s sleep quality and the next-day activity diversity: for the oldest-olds (≥80), higher sleep quality predicted greater activity diversity, whereas for the younger-olds (≤73), the association was negative. These findings highlight the nuanced role of nightly sleep quality in shaping daily activity participation across different age groups.

